# Effect of family doctor contract services on patient perceived quality of primary care in southern China

**DOI:** 10.1186/s12875-020-01287-7

**Published:** 2020-10-24

**Authors:** Shanshan Feng, Aiyun Cheng, Zhenni Luo, Yao Xiao, Luwen Zhang

**Affiliations:** 1 School of Health Management, Guangzhou Medical University, Xinzao Town, Panyu District, Guangzhou, 5111436 Guangdong People’s Republic of China; 2 School of Health Management, Southern Medical University, No. 1023-1063, South Shatai Road, Baiyun District, Guangzhou, 510515 Guangdong Province People’s Republic of China

**Keywords:** Primary care, Family doctor contract services, Quality, Primary care assessment tool (PCAT)

## Abstract

**Background:**

Family doctor contract service is an important service item in China’s primary care reform. This research was designed to evaluate the impact of the provision of family doctor contract services on the patient-perceived quality of primary care, and therefore give evidence-based policy suggestions.

**Methods:**

This cross-sectional study of family doctor contract service policy was conducted in three pilot cities in the Pearl River Delta, South China, using a multistage stratified sampling method. The validated Primary Care Assessment Tool-Adult Edition (PCAT-AS) was used to measure the quality of primary care services. PCAT-AS assesses each of the unique characteristics of primary care including first contact, continuity, comprehensiveness, coordination, family-centeredness, community orientation, culture orientation. Data was collected through face-to-face interviews held from July to November, 2015. Covariate analysis and multivariate Linear Regression were adopted to explore the effect of contract on the quality of primary care by controlling for the socio-demographic status and health care service utilization factors.

**Results:**

A total of 828 valid questionnaires were collected. Among the interviewees, 453 patients signed the contract (54.7%) and 375 did not (45.3%). Multivariate linear regression showed that contracted patients reported higher scores in dimensions of PCAT total score (β = − 8.98, *P* < 0.000), first contact-utilization(β = − 0.71,*P* < 0.001), first contact-accessibility(β = − 1.49, *P* < 0.001), continuity (β = 1.27, *P* < 0.001), coordination (referral) (β = − 1.42, *P* < 0.001), comprehensiveness (utilization) (β = − 1.70, *P* < 0.001), comprehensiveness (provision) (β = − 0.99, *P* < 0.001),family-centeredness(β = − 0.52, *P* < 0.01), community orientation(β = − 1.78, *P* < 0.001), than un- contracted after controlling socio-demographic and service utilization factors. There were no statistically significant differences in the dimensions of coordination (information system) (β = − 0.25, *P* = 0.137) and culture orientation (β = − 0.264, *P* = 0.056) between the two both groups.

**Conclusions:**

This study demonstrates that the pilot implementation of family doctor contract services has significantly improved patients’ perceived primary care quality in the pilot cities, and could help solve the quality problem of primary care. It needs further promotion across the province.

## Background

It has been proven worldwide that a strong primary care delivery system is the foundation and guarantee of an efficient health care system, and an increasing number of countries are taking measures to strengthen their primary care capacity [1, 2]. The leading five developing countries, BRICS countries, have all considered primary care as the foundational basis to achieve “Health for All” [3]. China’s health care system reform of 2009 has given more attention and resources to the primary care system [4, 5]. There are three levels of medical organizations providing health care in China: primary care facilities, secondary and tertiary hospitals. The primary health facilities refer to community health centers (CHC) and community health stations (CHS) in urban areas, as well as township hospitals and village clinics in rural areas. The primary care facilities are grassroots institutions providing both medical services and public health services to community residents [6]. A basic public health services program for all was implemented in 2011. The policy was that all primary care institutions should provide a free basic public health services package for all residents including establishing health records, physician examination for elder, elderly care, chronic disease follow-ups, and more [7]. However, as the service items were provided by specific provider teams, service provision was inconsistent and information was stored separately in segmented sectors. For example, after nurses had collected information from chronic condition follow-ups, there was no channel for them to transfer the information to clinical physicians. Clinical physicians could neither access health records, nor use the information to support clinical diagnosis. The separation of medical services and basic public health services hindered the quality of primary care and slowed its development. Some studies pointed out that the fragmented delivery of basic public health services has become a bottleneck in the improvement of quality of primary care in China [6, 8].

Evidence from many countries shows that the contract between physician and patient could improve the quality of primary care, and patients who have their usual source of care from their primary care doctors benefit most in health outcomes [9, 10]. Pilot trials implementing family doctor contract services were established in some regions in China in 2013 [11]. This trial was determined to integrate the provision of basic medical services and basic public health services in primary care institutions, and provide patient-centered care to residents. Under the contracted service model, residents could choose to contract with the preferred general physician team that is usually composed with general physicians, nurses, public health doctors. The contracts generally last for 1 year, and the residents can change the contracted doctors if they are not satisfied with the service for the next year [12]. The teams provide freely services package included: to create and manage individual health records, to treat common diseases, to give annual health examinations, and conduct proactive life intervention measures to prevent and manage chronic conditions, follow-ups, and referrals. However, treatment of common diseases was not free. The contracted residents were encouraged to establish stable connection with their GP and visit their GP when they have health problems, but patients still reserved freedom of choosing preferred medical facilities. The contracted GP teams receive reimbursements by capitation from public health funds, social insurance pool and government directions.

The provision of family doctor contract services should improve community members’ disease prevention, treatment and management. The contract between physician and patient is intended to strengthen the connection and communication between the physician teams and patients and their families, transform the former primary health center-based service relation into a real patient-centered service model, and establish stable physician-patient relationships. The contracted family doctor team, compared with the whole clinic, is seen as a more independent, flexible and loyal unit that could provide more accessible, continuous, coordinated and comprehensive services to their contracted patients. Starfield and Shi thought that the basic functions of primary care -accessibility, comprehensiveness, continuity, and coordination, affect each other and work together, and their synergies form a mechanism for promoting health at the primary level [13]. Accordingly, international society has achieved consensus to evaluate the quality of primary care from the level of realization of primary care functions [14–16].

The pilot trials were initiated in areas where the primary care system was solid and stable. Guangdong province was chosen, with pilot settings of family doctor contract services in the Pearl River Delta, China’s most developed delta, in cities that ranked first in providing qualified primary care services. The services were contracted and provided by physician teams working in primary care facilities. The physician teams were encouraged to develop the best service delivery that adapts to local circumstances.

A number of research projects have been carried out to evaluate the effect of family doctor-patient contract, but most of them have focused on the comparison of contracted and un-contracted patients on, for example, the effects of self-management, or the corresponding patient satisfaction and treatment compliance [17–19]. There has been little research that has explored the family doctor contract services on the overall quality of primary care [13]. This study evaluates the impact of the provision of family doctor contract services on the patient-perceived quality of primary care in terms of accessibility, comprehensiveness, continuity, coordination, to provide evidence to support the policy improvement of the contracted service.

## Methods

### Study design and sample

This cross-sectional study was conducted in the Pearl River Delta, Guangdong Province, China from July to November 2015. A multi-stage sampling method was used to choose samples. In the first stage, three cities were randomly chosen to perform the investigation from six pilot study sites that conducted family doctor contract services in the Pearl River Delta: Guangzhou City, Huizhou City and Jiangmen City. In the second stage, four CHCs were randomly chosen from each city’s urban districts. Two township hospitals were randomly chosen from each city’s rural areas, and then two village clinics were randomly chosen from each township hospital’s management areas. Since Guangzhou is the only metropolitan area among the three study sites, eight CHCs, four township hospitals and eight village clinics (VCs) were chosen to keep the sampling rational. In total, 16 CHCs and 16 VCs were chosen to conduct the investigation. In the third stage, 30 study participants from sample CHC and VC were selected. Nine hundred sixty patients took part in the investigation, and 828 valid questionnaires were collected. The inclusion criteria were as follows: (1) study participants should be local residents who were over 18; (2) study participants must sign a consent form and be able to understand the content of the questionnaire; (3) study participants must have visited the study sites at least once in the past year. Ethical approval was obtained from the Ethics Committees of Guangzhou Medical University.

This sample size was determined based on findings from existing published literature. Current research shows that a minimum sample size for such an analysis is 800, with a 99% confidence interval and a power of 80%. A minimum sample size of 300 for each setting was considered necessary to conduct the comparative analysis [20–23]. In this study, the sample size was 828, and the sample size for each group was above 375, which was regarded adequate to provide good statistical power.

### Data collection

We recruited 14 graduate students and undergraduate from the School of Health Management of Guangzhou Medical University as investigators, who were good at Cantonese and Mandarin. The investigators were trained on the survey skills, including how to explain the purpose of the study and items of the questionnaire to the study subjects, in order to guarantee completeness and consistency of the survey.

The survey was conducted at community health centers (CHC) or village clinics (VC). We investigated the eligible patients who visited the facilities, and the survey ended when there were 30 patients. Since the respondents were mostly older people with low education, face-to-face interview was adopted. The investigator explained the purpose of the study and asked questions one by one. Then filled in the questionnaire based on the answers, and a small gift was given as a token of appreciation for the participation.

### Measures

The questionnaire had three parts -socio-demographic information, state of contract and service utilization, as well as the Adult Short Primary Care Assessment Tool (PCAT-AS).

Due to the current trend of measuring the functions of primary care by using patients’ perceptions of quality, this research adopted the internationally reputed assessment tool PCAT-AS as the major part of the questionnaire. PCAT was designed and developed by Dr. Barbara Starfield and Dr. Leiyu Shi in Johns Hopkins University, and has been used in a number of studies in different countries [24–28]. We received consent from JHU to use and adapt the questionnaire. The adapted Chinese version of the PCAT-AS questionnaire has been proven valid and reliable in previous studies [22, 29]. In this study, the reliability and validity of the scale were tested. The results showed that Cronbach’s α was 0.891 and KMO was 0.879(Bartlett = 957.483, *P* = 0.000), the scale has good reliability and validity. The PCAT-AS measures four core functions of primary care, namely, first-contact (accessibility and utilization), continuity, coordination (information-system and referral, coordination), comprehensiveness (utilization and provision), and three expand functions of primary care, namely, family-centeredness, community-orientation and culture-orientation. Since 3 out of 4 core functions contain two sub-dimensions, a total of 10 dimensions are included here. The four-point Likers scale was adopted, with one point representing ‘definitely not’ four points ‘definitely yes’, and nine points ‘not sure’. Higher scores indicate better patient perception of primary care quality [30].

We used the question “Have you signed a contract with a physician /physician team in CHCs or VCs?” to define the state of contract. ‘Yes’ would be the contracted and ‘no’ would be un-contracted. The questionnaire also included socio-economic information like age, sex, marriage, education, monthly income, and use of health service information like self-assessed health status, satisfaction with services and primary care institutions, chronic conditions, and the percentage of medical care expenditures to total consumption.

### Statistical analysis

The questionnaires were double checked immediately after the interviews. Data documentation was done with Epidata 3.1 by two independent students and a cross-check was made after data input. Software 18.0 was used to perform the analysis.

Chi-square tests were conducted to compare the socio-demographic characteristics and health care utilization of participants between the group of contracted service patients and group of un-contracted service patients. Covariate analysis was adopted to compare the PCAT scores of the two groups. Then we compared the adjusted PCAT scores by analysis of covariance between the two groups. Lastly, multivariate linear regression was done to explore the effect of contract on the quality of primary care by controlling for the other influencing factors. In this analysis, the multivariate linear regression was performed by using each dimension as well as PACT total scores as dependent variables, the reception of contract services as independent variable, and the socio-demographic (sex, age, education, marital status, family income) and service utilization factors (self-evaluated physical health, patients’ satisfaction, proportion of medical expenditure to total family expenditure, chronic conditions) as control variables. Stepwise regression was used with *P* < 0.05 as inclusion criteria, and *P* > 0.10 as exclusion criteria. Multivariate linear regression analysis requires a sample size of more than 20times the number of independent variables. In this study, the number of independent variables in the multiple linear regression analysis is 10 variables, and the sample size is 828 greater than 200. A *p*-value< 0.05 was considered statistically significant in the analysis.

## Results

A total of 960 patients were approached for study inclusion, 828patients responded to the questionnaire, and 132 patients did not complete investigation. This response rate was 86.25%. The most common reason for un-response was having no time. Since the questionnaire was collected through face-to-face interviews, the completed questionnaire had almost no missing values. The average age was 61.9. Over 60% of patients were over 60. Of the interviewees, 453 patients had signed the contract (54.7%) and 375 did not (45.3%). There were no significant differences in terms of gender, marital status, self-evaluated physical health and chronic conditions between the two groups (*p* > 0.05). However, significant differences were reported in the dimensions of age, education, family monthly income, patient satisfaction, and proportion of medical expenditure to total family expenditure (*p* < 0.05) (Table 1).

**Table 1:** The socio-demographic states and health care service utilization between the contracted and un-contracted patients

Variables	Scales	Total (NO. %; ***n*** = 828)	Contracted (NO.%; ***n*** = 453)	Un-contracted (NO. % ***n*** = 375)	χ2	***p***-value
Socio-demographic characteristics						
gender	Male	310 (37.4)	174 (38.4)	136 (36.3)	0.40	0.564
	Female	518 (62.6)	279 (61.6)	239 (63.7)		
Age	18–34	55 (6.6)	17 (3.8)	38 (10.1)	38.11	< 0.001
	35–59	237 (28.6)	102 (22.5)	135 (36.0)		
	≥60	536 (64.7)	334 (73.7)	202 (53.9)		
Marital status	Yes	150 (18.1)	80 (17.7)	70 (18.7)	0.14	0.718
	No	678 (81.9)	373 (82.3)	305 (81.3)		
Education	Junior high and lower	580 (70.0)	360 (79.5)	220 (58.7)	42.32	0.000
	Senior high and above	248 (30.0)	93 (20.5)	155 (41.3)		
Family monthly income	≥3000 RMB	532 (64.3)	320 (70.6)	212 (56.5)	17.77	0.000
	< 3000 RMB	296 (35.7)	133 (29.4)	163 (43.5)		
Service utilization						
Self-evaluated physical health	Good	283 (34.2)	149 (32.9)	134 (35.7)	0.73	0.418
	Fair and poor	545 (65.8)	304 (67.1)	241 (64.3)		
Patient satisfaction	Satisfied	621 (75)	354 (78.1)	267 (71.2)	5.28	0.024
	Unsatisfied	207 (25)	99 (21.9)	108 (28.8)		
Proportion of medical expenditures to total Family expenditures	< 10%	358 (43.2)	212 (46.8)	146 (38.9)	5.17	0.024
	10%以上	470 (56.8)	241 (53.2)	229 (61.1)		
Chronic conditions	yes	434 (52.4)	248 (54.7)	186 (49.6)	3.27	0.195
	no	393 (47.5)	205 (45.3)	188 (50.1)		

Since continuity referral was limited to patients who had been referred to large hospitals, only 45.2% patients who were qualified to answer within this dimension. To avoid possible bias, the calculation of PCAT total score excluded this continuity (referral) dimension. The average of PCAT score was 27.68. Covariate analysis was adopted to compare the two gro-up of patients’ perceived feeling of the quality of each dimension as well as the PCAT score controlling for the socio-demographic and service utilization factors. The adjusted score of each PCAT dimension were calculated, and *P* value was offered to show the difference between groups, after controlling for gender, age, marital status, education, family monthly income, self-evaluated physical health, patient satisfaction, proportion of medical expenditure to total family expenditure, and chronic conditions. The patients who received family doctor contract services reported higher scores than those that did not, in the dimensions of total PACT score (28.79 vs26.43;*p* < 0.001),first contact- utilization (3.40vs3.17; *p* < 0.001), first contact accessibility (3.00vs2.63;*p* < 0.001),continuity (3.03 vs2.71; *p* < 0.001), coordination (referral) (3.17vs2.82,p0.001), comprehensiveness (service utilization)(3.40vs3.06;*p* < 0.001), and comprehensiveness (service provision)(3.50vs 3.25;*p* < 0.001);family-centeredness (2.87vs2.69;*p* < 0.01), community orientation (2.99vs2.40;*p* < 0.001), No significant difference was found in coordination (information system) and culture orientation between the two groups of patients (Table 2).

**Table 2:** Primary care quality scores between contracted and un-contracted patients, adjusted

Dimensions	Adjusted-mean(95%CI)			***p***-value
	contracted (1)	Un-contracted (2)	D-value (1)–(2)	
First contact-utilization	3.40 (3.35,3.46)	3.17 (3.11,3.23)	0.23	< 0.001
first-contact-accessibility	3.00 (2.95,3.07)	2.63 (2.56,2.70)	0.37	< 0.001
continuity	3.03 (2.97,3.09)	2.71 (2.64,2.78)	0.32	< 0.001
coordination (referral)	3.17 (3.07,3.28)	2.82 (2.70,2.94)	0.35	< 0.001
coordination	3.32 (3.25,3.39)	3.23 (3.15,3.31)	0.09	0.137
(information system)				
comprehensiveness	3.40 (3.35,3.46)	3.06 (3.00,3.12)	0.34	< 0.001
(utilization)				
comprehensiveness	3.50 (3.43,3.57)	3.25 (3.18,3.32)	0.25	< 0.001
(provision)				
family-centeredness	2.87 (2.79,2.94)	2.69 (2.61,2.78)	0.18	0.003
community orientation	2.99 (2.93,3.05)	2.40 (2.33,2.47)	0.59	< 0.001
culture orientation	3.26 (3.20,3.32)	3.17 (3.11,3.24)	0.09	0.056
total score	28.79 (28.45,29.14)	26.34 (25.96,26.72)	2.45	< 0.001

Notes: Nine dimensions were included to calculate the total score. The dimension of coordination (referral) was excluded as it was only answered by a few patients who received referral services

Further analyses were made to explore the effect of the contract on the quality of primary care by controlling for the other influencing factors. The multivariate liner regression results showed that the patients who received contract services reported higher PCAT total scores (β = − 8.98, *P* < 0.000),first contact-utilization(β = − 0.71, *P* < 0.001), first contact-accessibility(β = − 1.49, *P* < 0.001), continuity(β = 1.27, *P* < 0.001), coordination (referral) (β = − 1.42, < 0.001), comprehensiveness (utilization) (β = − 1.70, *P* < 0.001), comprehensiveness (provision) (β = − 0.99, *P* < 0.001),family-centeredness(β = − 0.52, *P* < 0.01),community orientation(β = − 1.78, *P* < 0.001), than those who did not receive contract services after controlling for socio-demographic and service utilization factors. There were no statistically significant differences in the coordination (information system) (β = − 0.25, *P* = 0.137) and culture orientation (β = − 0.264, *P* = 0.056). (Table 3). In addition, factors significantly associated with higher PCAT total scores included patient satisfaction and chronic conditions. Patients satisfied with primary care institutions and those with chronic conditions tended to report better primary care experiences.

**Table 3:** Multivariate liner regression of the PACT scores

**variable**	**total score** **β (95% CI)**	**first contact-utilization** **β (95% CI)**	**first contact-accessibility** **β (95% CI)**	**continuity** **β (95% CI)**
Contract				
yes				
no	−8.98(−10.83,-7.13)***	−0.71 (0.96,0.45) ***	−1.49(− 1.87,-1.12) ***	−1.27(− 1.63.-0.90)***
socio-demographic				
gender				
male (ref)				
female	−.906(−2.76,0.94)	0.03(−0.23,0.28)	−0.44(− 0.81,-0.06) *	− 0.15(− 0.52,0.22)
age				
18–59(ref)				
≥ 60	−1.335(−3.42,0.75)	0.12(−.17,040)	− 0.40(− 0.82,0.03)	−0.41(− 0.82,0.01)
Marital status				
Unmarried (ref)				
married	1.31(−.99,3.61)	−.20 (0.34,0.29)	0.20(− 0.27,0.67)	−0.39(− 0.07,0.85)
education				
Junior high and lower (ref)				
Senior high and above	−0.26(−1.38,0.84)	− 0.22(− 0.37,-0.07)**	−0.18(− 0.41,0.04)	−0.24(− 0.46,-0.01) *
Family monthly income(¥)				
< 3000(ref)				
≥ 3000	−0.25(−1.24,0.74)	− 0.17(− 0.31,-0.03) *	−0.32(− 0.52,-0.12) **	− 0.17(− 0.37,-0.03)
Service utilization				
Self-evaluated				
Good (ref)				
Fair and poor	−0.39(− 1.37,0.60)	0.04(− 0.09,-.18)	− 0.07(− 0.27,0.13)	0.01(− 0.19,0.21)
Patient’s satisfaction				
Satisfaction (ref)				
Un-satisfaction	−9.25(− 11.30,-7.20)***	−0.78(− 1.06,-0.50)***	− 1.27(− 1.70,-0.86) ***	−1.37(− 1.78.-0.96)***
Proportion of medical				
Expenditures to total family expenditures				
≤ 10%(ref)				
> 10	0.66(−1.21,2.52)	−0.12(− 0.38,0.13)	0.37(− 0.01,0.75)	0.05(− 0.32,0.43)
Chronic conditions				
no (ref)				
Yes	2.53 (1.00,4.06) **	0.20(−0.10,-.41)	− 0.04(− 0.35,0.26)	0.42 (0.11,0.72) **
**variable**	**Coordination (referral)** **β (95% CI)**	**Coordination (information system)** **β (95% CI)**	**Comprehensiveness (utilization)** **β (95% CI)**	**Comprehensiveness (provision)** **β (95% CI)**
Contract				
yes				
no	−1.42(− 2.09,0.74)***	−0.25(− 0.58,-0.08)	− 1.70(− 2.11,-1.29)***	−1.70(− 2.11,-1.29)***
socio-demographic				
gender				
male (ref)				
female	−0.30(− 0.98,0.37)	0.25(− 0.08,0.58)	− 0.33(− 0.73,0.08)	− 0.22(− 0.62,0.18)
age				
18–59(ref)				
≥ 60	0.26(−0.51,1.04)	− 0.08(− 0.46,0.29)	−0.08(− 0.46,0.29)	0.05(− 0.40,0.50)
Marital status				
Unmarried (ref)				
married	−0.20(− 0.99,0.59)	0.41(− 0.01,0.82)	− 0.22(− 0.73,0.08)	−0.05(− 0.54,0.45)
education				
Junior high and lower (ref)				
Senior high and above	−0.42(− 0.82,-0.02)	− 0.24 (0.04,0.44) **	0.16(− 0.09,0.40)	−0.09(− 0.33,0.15)
Family monthly income(¥)				
< 3000(ref)				
≥ 3000	−0.05(− 0.40,0.30)	0.12(− 0.06,-.30)	0.14(− 0.08,0.36)	0.14(− 0.08,0.35)
Service utilization				
Self-evaluated				
Good (ref)				
Fair and poor	−0.18(− 0.18,0.54)	0.01(− 0.17,0.18)	−0.09(− 0.31,0.13)	− 0.15(− 1.60,-0.71)
Patient’s satisfaction				
Satisfaction (ref)				
Un-satisfaction	−0.79(− 1.53,-0.05)	−0.65(− 1.02,-0.28) **	−1.17(− 1.62,-0.72) ***	−1.15(− 1.60,-0.71) ***
Proportion of medical				
Expenditures to total family expenditures				
≤ 10%(ref)				
> 10	0.03(− 0.71,0.76)	0.25(−0.09,0.58)	− 0.51(− 0.92,-0.10) *	0.21(− 0.19,0.61)
Chronic conditions				
no (ref)				
Yes	− 0.43(− 1.18,0.32)	0.39 (0.11,0.66) **	0.53 (0.19,0.87) **	0.49 (0.16,0.82) **
**variable**	**Family-centeredness** **β (95% CI)**	**Community-orientation** **β (95% CI)**	**Culture-orientation** **β (95% CI)**	
Contract				
yes				
no	−0.52(− 0.86,-0.18) **	−1.78(−2.06,-1.49) ***	− 0.26(− 0.54,0.01)	
socio-demographic				
gender				
male (ref)				
female	−0.03(− 0.37,-.31)	− 0.30(− 0.58,-0.01) **	0.28 (0.01,0.55)	
age				
18–59(ref)				
≥ 60	−0.35(− 0.74,0.03)	− 0.35(− 0.74,0.03)	−0.19(− 0.49,-.12)	
Marital status				
Unmarried (ref)				
married	0.08(−0.34,0.50)	0.13(−0.23,0.48)	0.40 (0.06,0.73) *	
education				
Junior high and lower (ref)				
Senior high and above	0.11(−0.09,0.31)	− 0.08(− 0.25,0.09)	0.04(− 0.124,0.20)	
Family monthly income(¥)				
< 3000(ref)				
≥ 3000	−0.05(− 0.23,-.14)	−0.02(− 0.17,0.14)	0.08(− 0.07,0.23)	
Service utilization				
Self-evaluated				
Good (ref)				
Fair and poor	−0.13(− 0.32,-.04)	−0.04(− 0.19,-.12)	0.04(− 0.11,0.18)	
Patient’s satisfaction				
Satisfaction (ref)				
Un-satisfaction	−0.93(− 1.30,-0.55) ***	−1.16(− 1.48,-0.84) ***	−0.77(− 1.07,-0.47) ***	
Proportion of medical				
Expenditures to total family expenditures				
≤10%(ref)				
> 10	0.47 (0.13,0.81) **	−0.06(− 0.35,0.23)	0.01(− 0.27,0.28)	
Chronic conditions				
no (ref)				
Yes	0.31 (0.03,0.59) *	0.04(−0.20,0.27)	0.20(−0.02,0.42)	

***p < 0.001

**p < 0.01

*p < 0.05

## Discussion

This research has explored the association between family doctor contract services and patient perception of primary care quality using an internationally developed tool in China. The results have shown that the physician/patient contracted service delivery model could significantly improve the perceived quality of primary care after controlling socio-demographic and service utilization factors.

A research made in the East European countries has showed that the barrier lays in the primary care system in the East European countries is the lack of cooperation among medical disciplines and sectors [31]. Yuan et al. finds that the quality of primary care is influenced by the hampered cooperation among the basic clinical and public health departments in China [6]. It is important that public health services and primary care services offered by the same team, to improve the quality of primary care and chronic condition management by improving the continuity of care [6]. This physician-patient contract policy promoted to reorganize the current fragmented medical care and basic public health service delivery system in primary care facilities, and improve the quality of primary care. It results in substantial changes in patients’ perceptions of primary care quality. A previous study conducted in Montenegro has shown that the primary care reform in Montenegro had been proven significant improvement in primary care use and patients’ satisfaction. The reform included patient’s compulsory contraction with GP, GP’s obligation of offering medical and public health services, as well as the support to establish GP team [32].

This study has shown that patients that received family doctor contract service reported higher scores than those that did not receive the service, in the dimensions of first contact and continuity. Continuity care refers to the longitudinal use of a regular source of care over time [30]. A previous study has shown that the contracted residents reported a higher rate of choosing primary care institution as the first contact compared with the un-contracted residents [17]. For the contracted physicians the contract enhanced their sense of responsibility to the contracted patients and improved doctor-patient communication. This behavior change in physicians could restore mutual trust, improve patient loyalty and establish long-term relationship [33, 34]. Linni Gu’s research has shown that the contracted can improve the communication between physicians and patients, which would contribute to the mutual trust [35]. This indicates that the physician-patient relationship is strengthened by the service contract. In addition, the contract service could improve continuity through offer better integration of basic public health and medical services in primary care institutions. A study conducted in Quebec, Canada has shown that the primary care reform which aimed to promote interdisciplinary collaborative practices improved patient’s experience in continuity of care [36]. Therefore, more patients would use the contracted physician’s office as the usual source of care, so the scores of first contact and continuity dimensions would improve as a consequence [32]. The ongoing doctor-patient relationship is the core spirit of primary care [32, 37]. Through knowing patients’ medical history and health status, doctors could offer patients personalized care and could diagnose diseases in the early stages. Patients could show better compliance and efficacy.

The high score of contracted patients in the coordination (referral) could be explained by the contract items required the contracted primary care institutions to provide referral services to contracted patients, which improved referral services. This study has shown that the patients who received family doctor contract service reported higher scores than those did not receive the service, in dimensions of comprehensiveness. Comprehensive care refers to the availability of a wide range of health services in primary care and their appropriate provision across the entire spectrum of types of health needs [30]. The contract service list also covers basic public health care services and medical services, which has ended the history of fragmented public service delivery system and provided a comprehensive service package, including the establishment of individual health records, common disease treatment, chronic condition follow-up, and so on. This integration can improve the treatment effect by improving patient’s compliance [29]. These service items are offered to patients by contracted physicians and their team members, so that mutual connections were strengthened and it were helpful to improve the patients’ perceptions of comprehensiveness care.. Therefore; the contracted patients reported higher score in the dimension of comprehensiveness. With the increasing epidemic of chronic conditions, primary care should take the responsibility of offering more comprehensive services [31]. The most effective way to attain this goal is to expand the contraction between residents and GP teams.

In addition, this study demonstrates that contracted patients are more likely than un-contracted patients to rate their primary care as good in dimensions of family-centeredness and community orientation. With the advance of interpersonal communication and stable relationship, the contracted physicians could learn more about patients’ family conditions, community characteristics, and consider these factors when treating patients. As a result, the contract patients’ evaluation of the dimensions of family centered-ness, community orientation, was higher than the un-contracted patients.

Analysis of the scores of each dimension showed that patients did not report differences in the dimensions of coordination (information system) and culture orientation. Coordination (information system) means the capacity of connecting all sides of information service provision participants as well as the service process [34]. Regardless of the contracted or un-contracted residents, the information is recorded and transferred in the same way, so there is no difference between the both groups in terms of coordination (information). The culture dimension refers to the respect to patients’ religion, values or specific behaviors that may influence health outcomes. Contract patients could have a different perception of cultural respect due to their stronger relationship with health providers. Maybe the difference will be more obvious after a long time.

This research had some limitations. First, this research explored the functional adequacy offered to contracted residents under the primary care functional theory; however, it may neglect the description of the objective quality improvement brought by contract services. Subjective indicators will be adopted as well in the further study. Secondly, there might be bias caused by local residents who refused to be interviewed the most common reason for un-response was having no time, and this study did not collect information of residents who refused to take the interview. However, the face-to-face interview increased the response rate to 86.25%, which might be higher than collecting data by mail, phone interview or self-response and could correct the bias to some extent. Future studies we should compare participants’ characteristics with those of patients who did not participant. Thirdly, this is a cross-sectional survey which may not have explored causal relationship from these findings.

## Conclusions

In conclusion, the implementation of family doctor contract service has generally improved primary care quality, which is worth further promotion provincially or even nationally. This is a cross-sectional study, but longitudinal research should be made accordingly to improve the accuracy of the evaluation and explore the influencing mechanism.

## Data Availability

The datasets used and analyzed during the current study available from the author on reasonable request (send email to: fengshsh@126.com).

## Abbreviations

PCAT-AS: Primary Care Assessment Tool-Adult Edition
PCAT: Primary Care Assessment Tool
CHCs: Community Health Centers
VCs: Village clinics
